# Do the A Subunits Contribute to the Differences in the Toxicity of Shiga Toxin 1 and Shiga Toxin 2?

**DOI:** 10.3390/toxins7051467

**Published:** 2015-04-29

**Authors:** Debaleena Basu, Nilgun E. Tumer

**Affiliations:** Department of Plant Biology and Pathology, School of Environmental and Biological Sciences, Rutgers University, New Brunswick, NJ 08901-8520, USA; E-Mail: debabasu@rutgers.edu

**Keywords:** shiga toxin, ribosome inactivating protein, P-protein stalk binding, sarcin/ricin loop depurination

## Abstract

Shiga toxin producing *Escherichia coli* O157:H7 (STEC) is one of the leading causes of food-poisoning around the world. Some STEC strains produce Shiga toxin 1 (Stx1) and/or Shiga toxin 2 (Stx2) or variants of either toxin, which are critical for the development of hemorrhagic colitis (HC) or hemolytic uremic syndrome (HUS). Currently, there are no therapeutic treatments for HC or HUS. *E. coli* O157:H7 strains carrying Stx2 are more virulent and are more frequently associated with HUS, which is the most common cause of renal failure in children in the US. The basis for the increased potency of Stx2 is not fully understood. Shiga toxins belong to the AB_5_ family of protein toxins with an A subunit, which depurinates a universally conserved adenine residue in the α-sarcin/ricin loop (SRL) of the 28S rRNA and five copies of the B subunit responsible for binding to cellular receptors. Recent studies showed differences in the structure, receptor binding, dependence on ribosomal proteins and pathogenicity of Stx1 and Stx2 and supported a role for the B subunit in differential toxicity. However, the current data do not rule out a potential role for the A_1_ subunits in the differential toxicity of Stx1 and Stx2. This review highlights the recent progress in understanding the differences in the A_1_ subunits of Stx1 and Stx2 and their role in defining toxicity.

## 1. Introduction

Shiga toxin producing *Escherichia coli* (STEC) strains such as *E. coli* O157:H7, as well as other serotypes, are the major causative agents of severe gastroenteritis, which can lead to life-threating complications including hemorrhagic colitis (HC) and hemolytic uremic syndrome (HUS) [1,2]. HUS is the most common cause of renal failure in children in the US [3]. The recent multi-state outbreak of *E. coli* O157:H7 in the US and a HUS outbreak in Germany in 2011 caused by *E. coli* O104:H4 highlight the public health impact of this pathogen [4,5,6,7]. STEC strains produce Shiga toxin 1 (Stx1) and/or Shiga toxin 2 (Stx2) or variants of either toxin. *E. coli* strains carrying Stx2 are more virulent and are more frequently associated with HUS [8,9,10]. However the molecular basis for the higher potency of Stx2 is unknown. Although extensive research is being undertaken to develop effective vaccines and therapeutics to protect against HUS, there are no current therapies available. In order to develop inhibitors against Shiga toxins, there is a need for better understanding of their underlying mechanism of toxicity.

Shiga toxin (Stx) from *Shigella dysenteriae* and Stx1 (Stx1) and 2 (Stx2) from Shiga toxin-producing *Escherichia coli* (STEC) are a family of structurally and functionally related proteins [5,11]. Stx, Stx1 and Stx2 are ribosome inactivating proteins (RIPs), a class of proteins that irreversibly damage the ribosome catalytically by modifying the large rRNA and inhibiting protein synthesis [12,13,14,15,16]. RIPs are present throughout the plant kingdom and are also found in bacteria [12,13,14]. RIPs are *N*-glycosidases that remove a specific adenine from the highly conserved α-sarcin/ricin loop (SRL) in the 28S rRNA of the large ribosomal subunit [12,13,14]. Irreversible modification of the target adenine blocks elongation factor (EF)-1- and EF-2-dependent GTPase activity and renders the ribosome unable to bind EF-2, thereby blocking translation [17,18]. The RIPs are divided into three types based on their physical properties. Type 1 RIPs such as pokeweed antiviral protein (PAP), trichosanthin (TCS) and saporin are single chain, highly basic monomeric enzymes, approximately 30 kDa in size [19,20,21] Type 2 RIPs consist of an A chain and variable number of B chains. The A chain is the active chain, while the B chain can bind receptors on the surface of eukaryotic cells and mediate retrograde transport of the A-chain to the cytosol. Potent toxins like ricin, abrin and Shiga toxins fall into this category. Type 3 RIPs are synthesized as inactive precursors (proRIPs) that require proteolytic processing to occur between the amino acids involved in formation of the active site [13,15]. Because of their potent and selective toxicity, RIPs have been exploited as potential agents of bioterrorism and have garnered interest for use in antiviral and anticancer therapy [14,22,23]. Shiga toxin and its B subunit have been investigated as novel therapeutic agents against pancreatic cancer and colon cancer [24,25].

## 2. Structure

Stx derives its name from the dysentery causing bacteria, *Shigella dysenteriae*, which was first described by Kiyoshi Shiga in 1898. While Stx from *S. dysenteriae* differs from Stx1 by one amino acid [26,27], Stx1 and Stx2 have only 56% amino acid similarity [28] and are antigenically distinct [28,29,30]. STEC can produce either one type of toxin or a combination of variants of one or both types of toxin [31]. Stx1 and Stx2, which are also referred to as Stx1a and Stx2a [32], are type II RIPs, which consist of a catalytically active A chain associated with a pentamer of B subunits responsible for the binding of the Shiga toxins to their common cellular receptor, globotriaosylceramide (Gb3) [33,34]. The B subunits (7.7 kDa each) form a central pore which harbors the *C*-termini of the A subunit [35]. The crystal structure of the Stx1 B subunit pentamer, bound with Gb3 shows that each B monomer contains three distinct binding sites for the glycan component of Gb3, referred to as P^k^ trisaccharide, α-d-Gal*p*-(1-4)-β-d-Gal*p*-(1-4)-β-d-Glc*p*-(1-O) for a total of 15 sites [36]. Of these three binding sites (labeled 1–3), site 2 has the highest occupancy of electron density defining the position of the trisaccharide, while site 1 has the lowest [36,37]. The only known exception to this Gb3-dependence of Shiga toxins is for the Stx2 variant Stx2e, which exhibits specific affinity for globotetraosylceramide (Gb4) [38,39], although Stx1 and Stx2 can bind to Gb4 weakly [40]. Stx2e has recently been shown to bind to the Forssman glycolipid, which makes this subtype unique among the Stx subtypes [41]. Recently, the crystal structure of Stx2 bound to a P^k^ derivative has been published [42]. This structure showed that only two of three previously identified binding sites on the B_5_ pentamer was functional in Stx2, indicating that there are differences in receptor binding between Stx1 and Stx2a [42].

The A subunit of Shiga toxin consists of A_1_ and A_2_ chains which are bound together by a disulfide bond between C242 and C261 forming a loop [43]. The X-ray crystal structures of *Shigella* Stx and Stx2 are highly similar [34,35]. However, structural differences have been identified between Stx1 and Stx2 [34,35]. In Stx1, part of the active site is blocked by the A_2_ chain, while it is accessible in Stx2 [35]. The active site of Stx2 is accessible to the adenine substrate and Stx2 cleaves the adenine when it is crystallized in the presence of adenosine [44]. In the crystal structure, the A subunit in Stx2 is in a different orientation with respect to the B subunit, which may affect receptor affinity of Stx2 [35]. The *C*-terminus of Stx2 extends through the pore formed by the B pentamer, which is thought to interfere with receptor binding [35]. However, it is not known if the A subunits contribute to the interaction of the holotoxins with the receptor and whether the A subunit interferes with Gb3 binding. Stx1 and ricin have been shown to interact with human neutrophils, which do not express Gb3 or Gb4, through their A subunit without inducing their internalization [45]. TLR4 has recently been identified as the receptor that recognizes the A subunits of Stx1 and Stx2 in human neutrophils [46].

Once the toxins bind the globotriaosylceramide (Gb3) receptor, they are endocytosed by a clathrin-dependent or independent pathway [11,47]. They then undergo retrograde transport to the Golgi apparatus and then to the endoplasmic reticulum (ER). The active A_1_ subunit is cleaved enzymatically from the A_2_-B5 complex [43]. The cleavage occurs between R251 and M252 in Shiga toxin and Stx1 and between R250 and A251 in Stx2 by the furin protease. After cleavage, the A_1_ fragment remains bound to the A_2_ fragment through the disulfide bond. The A_1_ chain is then released from the A_2_-B5 complex by reduction of the disulfide bond in the ER and undergoes retrotranslocation from the ER into the cytosol where it depurinates the ribosome and inhibits protein synthesis [11,47].

## 3. Catalytic Activity and Cytotoxicity of Stx1 and Stx2

### 3.1. Differences in Cytotoxicity

An extensive review on the pathophysiology of Stx-related disease in different animal models can be found in [48] and is briefly described here. Epidemiological studies suggest that majority of the HUS-associated fatalities are caused by *E. coli* O157:H7 strains carrying Stx2 [8,9,10]. Previous studies using Shiga toxins have shown that while Stx2 is more potent in animal models, Stx1 is more toxic to Vero cells [49,50]. The 50% lethal dose for purified Stx2 was 400-fold lower than for Stx1 in a mouse model, and only Stx2-treated mice developed renal complications and death [49,51]. However, animal models have limitations compared with the observations from humans and do not replicate the disease in humans. Nonhuman primate models (Baboon) showed renal damage consistent with HUS upon intravenous injection of the toxins. Treatment of non-human primates with four doses of 25 ng/kg Stx2 caused HUS, while an equal dose of Stx1 had no effect [50]. In another study comparison of the effects of the two toxins showed interesting differences, including different proinflammatory responses and different timings with delayed organ injury after Stx2 challenge [52]. Baboons treated with Stx1 developed HUS within two to three days, while those with Stx2 took longer (3–5 days), indicating the role of other factors in producing delayed renal injury upon challenge by Stx2. Furthermore, Stx1 incited a stronger proinflammatory response earlier, while the proinflammatory response induced by Stx2 was gradual and delayed by several days [52]. A subsequent study using baboon models showed that both Stx1 and Stx2 can affect kidney function. Although Stx2 was found to cause more severe damage to the kidney than Stx1, the damage inflicted on the kidney by Stx1 was significant [53].

In comparison to animal models studies in Vero cells suggested that the cytotoxicity of Stx1 is 10-fold greater than Stx2 [49]. The basis for the differential toxicity of Stx1 and Stx2 in animal models *versus* mammalian cell lines is unknown. Shiga toxins trigger endothelial damage in kidney and brain by targeting Gb3. However, differences have been observed in the sensitivity of endothelial cells to Stx1 and Stx2. The current knowledge of endothelial cell damage caused by Stx1 and Stx2 is reviewed in [54]. Stx2 had a higher potency for human renal microvascular endothelial cells (HRMEC) than to human umbilical vein endothelial cells (HUVEC), where toxicity of Stx1 and Stx2 was similar [55]. These results indicated selective sensitivity of renal endothelial cells to Stx2 although the renal endothelial cells possessed fewer Gb3 receptor binding sites for Stx2 than Stx1 [55]. The Stx receptor distribution in the different renal cell populations and the sensitivity of the different kidney cell types to Stx1 and Stx2 is reviewed in [11]. Comparison of cellular injury induced by Stx1 and Stx2 in human brain microvascular endothelial cells (HBMEC) and HUVEC derived EA.hy 926 macrovascular endothelial cells indicated that these cell lines had differential susceptibility to the toxins. HBMEC cells were over 1000-fold more susceptible to Stx2, while EA.hy 926 cells were around 10-fold more susceptible to Stx1 [56]. Stx1 caused both necrosis and apoptosis, while Stx2 induced mainly apoptosis in both cell lines [56]. The basis for the differential susceptibility of endothelial cells to Stx1 and Stx2 is not well understood. Holotoxin stability, enzymatic activity and receptor affinity were proposed as potential factors contributing to the differential toxicity. In addition, the cytotoxicity comparisons between Stx1 and Stx2 in animals and cells are critically dependent on the specific batches of toxin used and can vary accordingly.

The B subunits of Stx1 and Stx2 have been hypothesized to play an important role in mediating the differences in potency. The B subunits of Stx1 and Stx2 display differences in receptor recognition, as well as in the number of potential binding sites [57,58]. Studies in Vero cells demonstrated that Stx1 has a higher affinity for the Gb3 receptor [49,59,60,61]. Using purified Gb3, it was shown that Stx1 has a 10-fold higher affinity for Gb3 compared to Stx2 [59]. It has been suggested that Stx1 might bind to Gb3 variants in the lung, preventing it from reaching more susceptible organs, such as the kidneys, whereas Stx2 binds preferentially to Gb3 variants in kidney. As a result, Stx1 shows decreased binding to kidney cells, which are the main targets for lethality in mice [62]. Analysis of binding kinetics to the glycolipid receptor analog using surface plasmon resonance (SPR) showed that Stx1 bound to the receptor analog better than Stx2 and had faster association and dissociation rates [40]. These results suggest that the differences in binding kinetics and affinity of the B subunits for the Gb3 receptor may be responsible for the greater toxicity of Stx1 to Vero cells.

The B subunits of Stx1 and Stx2 also display differences in structural stability [63]. The B pentamer of Stx1 was more stable than the B pentamer of Stx2 and bound the receptor with higher affinity than the B pentamer of Stx2 [63]. Moreover, while Stx1B subunits were able to bind glycolipids only as a stable pentamer, Stx2B subunits bound to glycolipids in lower oligomeric states [63]. These results suggested that differences in receptor affinity and receptor binding preferences may contribute to the differential toxicity of Stx1 and Stx2 by affecting their targeting to susceptible tissues.

Stx A/B subunit chimeras, where the A and B subunits of the two toxins have been interchanged, were used to study the contribution of the individual A and B subunits to toxicity [59]. The holotoxin as well as the chimeric toxins were used in mouse and in Vero cells to differentiate the roles of the subunits in toxicity [49,59]. However, the chimeric toxins were usually found to be less stable than the holotoxins due to incorrect folding [59] or showed equivalent cytotoxicity [64]. Chimeric toxins, created by operon fusions displayed cytotoxicity intermediate to Stx1 and Stx2 [37] or did not produce a functional chimera [65]. Therefore, clear conclusions regarding the role of each subunit in toxicity could not be deduced from these studies. A recent study used the A_2_ subunit along with the B subunit to increase the stability of the chimeric toxin [66]. The binding of the chimeric toxins to the Gb3 receptor and their translocation through the monolayers of the polarized HCT-8 cells were dependent on the origin of the B subunit, and the chimeric toxin with the Stx1B subunit had a higher affinity for the receptor than the Stx2B chimera. The toxicity of the chimeric toxins to Vero and HCT-8 cells indicated the importance of the origin of the B subunit although the B subunit accounted for less than 50% of the differential toxicity for Vero cells [66]. Perhaps, due to the instability of the chimeric toxins at pH 3, the oral administration of the chimeric toxin where the A subunit was from Stx1 in mice required at least 10 times more toxin as compared to native Stx2, while Stx1 or the chimeric toxin where the A subunit was from Stx2 failed to show any mortality in mice, even at a very high concentration. This study highlighted the importance of the B subunit in the differential toxicity of Stx1 and 2. The differential lethality in mouse was thought to take place at the level of toxicity to the kidney [66]. However, although *in vivo* results indicate that the B subunits are involved in differences in the severity of the intoxication, they do not rule out a potential role for the A subunits in the differential toxicity of Stx1 and Stx2. The critical question regarding why Stx2 is more potent than Stx1 *in vivo* still remains unanswered.

### 3.2. Differences in Catalytic Activity

The A subunits of Shiga toxins and ricin play a critical role in the toxicity of each toxin. They have the same catalytic activity and show conservation in amino acids at the active site. Mutagenesis studies identified Glu167, Arg170, Tyr77, Tyr114, Trp203 and Arg205, which are critical for the catalytic activity and are conserved between Stx1 and Stx2 [67,68,69,70,71,72]. Current knowledge about the mode of action of the A subunits is obtained from studies with either cultured cell lines or *in vitro* systems. *In vivo* studies at the molecular level or at the level of the whole organism are limited due to the extreme cytotoxicity of these toxins and the lack of available model systems. Using the yeast, *Saccharomyces cerevisiae* as a model, we identified the amino acids critical for the cytotoxicity of Stx1A and Stx2A and showed that the activity of the A subunits can be differentiated [70]. The results showed that Asn75 and Tyr77 were more critical for the depurination activity of Stx2A, while Arg176 was more critical for the depurination activity of Stx1A. Analysis of solvent accessible surface areas indicated that Asn75 and Tyr77 were more exposed in Stx2A, while Arg176 was more exposed in Stx1A [70]. Arg176 was subsequently shown to be critical for ribosome binding of Stx1A_1_ [73], suggesting that there may be differences in the ribosome binding of Stx1A_1_ and Stx2A_1_.

Several studies used cell-free translation inhibition assays to compare the enzymatic activity of the A subunits of Stx1 and Stx2 [49,50]. The A subunits displayed similar translation inhibitory activities [49,59,74], leading to the conclusion that the enzymatic activities of the A subunits are not responsible for the toxicity differences between Stx1 and Stx2. As a result the role of the A subunit in the differential potency of Stx1 and Stx2 has not received much attention. Since translation inhibition is a downstream effect of depurination, these assays did not directly compare the catalytic activity of Stx1A_1_ and Stx2A_1_ on the ribosome. Further, while in some studies the holotoxin was used [49], in others the holotoxin was activated by digestion with trypsin to release the A_1_ chain from the A_2_-B5 complex and/or by chemical treatment with DTT to break the disulfide bond between the A_1_ and the A_2_ chains [50,75]. These methods frequently yield variable amounts of activated protein and can cause degradation, preventing comparison of enzymatic activity directly [75]. Therefore, due to technical limitations, the role of the A_1_ subunit in increased potency of Stx2 has not been fully investigated and a direct comparison of the catalytic activity has not been carried out.

The unanswered questions regarding the relative catalytic activity of RIPs highlight the importance of quantitative assays, which allow direct comparisons of the depurination activity. Our group developed a quantitative real-time PCR (qRT-PCR) assay that can directly measure the catalytic activity of the RIPs on ribosomes *in vitro* or in yeast and in mammalian cells *in vivo* [76,77]. The qRT-PCR assay exhibited a much wider dynamic range than the previously used primer extension assay and increased sensitivity [76]. Sturm and Schramm described a quantitative enzyme coupled luminescence assay to examine the kinetics of depurination by RIPs [78]. In this assay, adenine released by depurination is converted to AMP by adenine phosphoribosyl transferase (APRTase) and then to ATP by pyruvate orthophosphate dikinase (PPDK). The light generated by ATP via firefly luciferase is detected using a luminometer [78]. The qRT-PCR and the enzyme coupled luminescence assay have been used to examine the activity of the ricin toxin A chain (RTA) and its mutants [79]. A highly sensitive and quantitative assay using SPR was developed by our group to examine the interactions RIPs with ribosomes [16,75,80,81]. The development of these assays will allow direct comparisons of the binding and depurination kinetics of the A_1_ subunits of Shiga toxins and will help determine whether the A_1_ subunits of Stx1 and Stx2 have a significant role in their differential toxicity.

## 4. Ribosome Interactions

Although the SRL is the primary substrate for all RIPs, ribosomal proteins play an important role in of the depurination of intact ribosomes by RIPs. While the *K_m_* of RTA for rat ribosomes and naked 28S rRNA are similar, RTA depurinates ribosomes almost 10^5^-fold greater than the naked 28S RNA [82], suggesting that not only the target RNA sequence, but also the structure of the ribosome plays a significant role in the catalytic activity of RIPs.

Previous studies have shown the importance of the ribosomal phosphoproteins (P) of the P-protein stalk for the depurination activity of the RIPs [73,75,80,81,83,84]. Ricin has been shown to crosslink to the stalk protein P0 and the ribosomal protein L9 [85]. Trichosanthin (TCS), which is a type-1 RIP, has been shown to interact with P0, and P1 proteins of the ribosomal stalk using yeast-two hybrid analysis and by *in vitro* binding assays [86]. The last 11 residues of P2, which are conserved in P0, P1 and P2 have been found to be critical for the interaction with trichosanthin (TCS) [87]. The crystal structure of TCS complexed to a peptide corresponding to the *C*-terminal domain (CTD) of human P2, SDDDMGFGLFD, showed that the conserved DDD motif at the *N*-terminal region of this peptide interacts with the positively charged K173, R174, and K177 residues in TCS, while the *C*-terminal region is inserted into a hydrophobic pocket [88]. Using yeast mutants deleted in the stalk proteins (ΔP1 and ΔP2) and highly sensitive SPR and depurination assays, our group provided the first evidence that the ribosomal stalk proteins are essential for the cytotoxicity of RTA *in vivo* and that the ribosomal stalk is the main landing platform for RTA on the ribosome [80]. We subsequently showed that multiple copies of the stalk proteins accelerate the recruitment of RTA to the ribosome for depurination [89].

The ribosomal P-protein stalk is a lateral flexible protuberance of the large ribosomal subunit, which recruits translational factors to the ribosome and participates in the GTPase activation by EF-Tu and EF-G. The eukaryotic P protein stalk consists of 11 kDa P1 and P2 proteins bound to a larger P0 protein. P1 and P2 dimerize via their helical *N*-terminal domains, whereas the highly conserved *C*-terminal tails of P1 and P2 interact with the translational GTPases (tGTPases) [90]. Although the stalk is relatively conserved in eukaryotes there are some notable differences between the stalk structure in mammals and in yeast. The human ribosomal stalk contains two identical heterodimers of P1 and P2 bound to P0 assembled into a pentameric complex [91,92]. In contrast the yeast pentameric stalk consists of four different proteins P1α, P1β, P2α, P2β [93] which form two different heterodimers [94], P1α-P2β and P2α-P1β, bound to P0 [95,96]. The human P1 has 40%–47% sequence identity with P1α and P1β and human P2 has 53%–56% sequence identity with P2α and P2β [97,98]. The prokaryotic equivalent of P1 and P2 are L12 proteins bound to a smaller P0 equivalent L10 [99]. In bacteria, the stalk structure can be a pentamer or heptamer [100], while in archaea the ribosomal stalk is a heptamer [101]. Although the eukaryotic and prokaryotic stalk proteins are analogous in function, there is no sequence homology between these related proteins [91,92].

**Figure 1:**
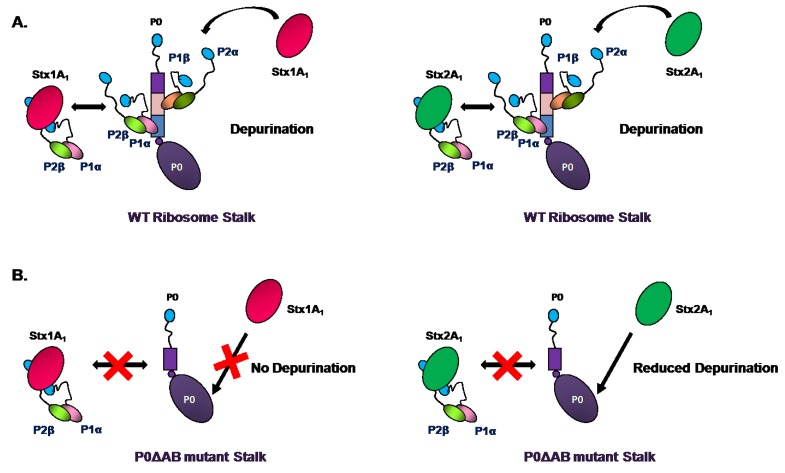
Model illustrating the interaction of Stx1A_1_ and Stx2A_1_ with the wild type and mutant stalk [75]. (**A**). Both Stx1A_1_ and Stx2A_1_ are able to interact with free P1α/P2β as well as ribosome bound P1α/P2β to depurinate the ribosome; (**B**). In the P0ΔAB mutant because the binding sites for P1/P2 dimers are deleted, free P1α/P2β proteins are not able to bind to the ribosomal stalk. Stx1A_1_ shows almost no depurination activity indicating its dependence on the ribosomal stalk. Stx2A_1_ has very little effect on depurination suggesting that it is less dependent on P1/P2 than Stx1A_1_.

Using the yeast-two-hybrid and pull-down experiments in HeLa cells, it was demonstrated that the A_1_ chain of Stx1 interacts with the P0, P1 and P2 proteins of the P-protein stalk [84]. Removal of the last 17 amino acids of P1 or P2, but not P0 abolished the interaction between Stx1A_1_ and the human ribosomal stalk proteins, suggesting that the conserved CTD of P1/P2 proteins allows Stx1 to access the SRL [84]. To determine if Stx1A and Stx2A require the ribosomal stalk for depurination *in vivo*, we examined their depurination activity and cytotoxicity in the yeast P protein deletion mutants [75]. Our results showed that ribosomal stalk is important for both toxins to depurinate the ribosome *in vivo*. Cytoplasmic stalk proteins were critical for Stx1A and Stx2A to access the SRL *in vivo* (Figure 1A). However, Stx1A and Stx2A differed in depurination activity towards ribosomes when P1/P2 binding sites on P0 were deleted. P1/P2 proteins facilitated depurination by Stx1A only if their binding sites on P0 were intact (Figure 1B). In contrast, Stx2A was less dependent on the stalk proteins for activity than Stx1A and could depurinate the ribosomes with a defective stalk better than Stx1A [75]. These results demonstrated that although ribosomal stalk is important for Stx1 and Stx2 to depurinate the ribosome, Stx2 is less dependent on the stalk proteins for depurination activity and suggested that cytosolic P1/P2 proteins deliver the toxins to the ribosome to create a toxin pool near the SRL [75].

The A_1_ chain of Stx1 was shown to interact with the ribosomal stalk proteins P0, P1, and P2 via the conserved CTD of P2 through hydrophobic and cationic surfaces on the toxin. Point mutations at arginines (Arg172, Arg176, Arg179, and Arg188) on Stx1A_1_ perturbed the interaction between the toxin and the P2 peptide [73]. Using a combination of SPR and yeast-two hybrid analysis, these arginines were shown to be critical for the interaction of Stx1A_1_ with the P2 peptide. The interactions with the P2 peptide were electrostatic and hydrophobic and took place at a site that was distinct from the active site. Since these residues are conserved between Stx1A_1_ and Stx2A_1_, it was proposed that Stx2 interacts with the ribosome in a similar manner [73].

**Figure 2:**
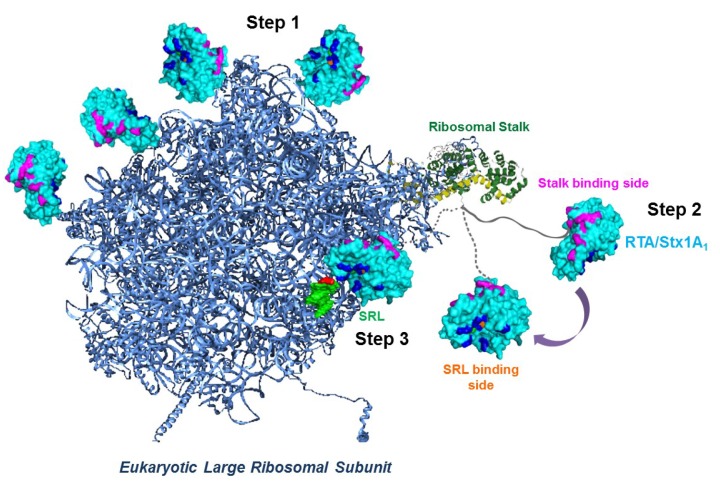
Model of how RTA and Stx1A_1_ may access the α-sarcin/ricin loop (SRL) of the large rRNA [79]. Eukaryotic large ribosomal subunit was created using Protein Data Bank (PDB) ID: 3U5I and Protein Data Bank ID: 3U5H (blue) using the PyMOL software (The PyMOL Molecular Graphics System, Version 1.3 Schrödinger, LLC) with the SRL (green). The fitted cartoon structure of P0 fragment complexed with the *N*-terminal domain of P-proteins (Protein Data Bank ID: 3A1Y) from archaea is depicted as yellow and green, respectively as described [79]. The flexible CTD domain of a P-protein is represented as a gray line. Ricin toxin A chain (RTA) (Protein Data Bank ID: 1RTC) is colored in cyan, its active site is shown in orange, RNA binding site in blue and the stalk binding site is shown in magenta. In Step 1 RTA/Stx1A_1_ are concentrated on the ribosome surface by nonspecific electrostatic interactions. In Step 2 RTA/Stx1A_1_ interact with the *C*-terminal domain (CTD) of P-proteins with their ribosome binding surface, which is on the opposite side of the surface that contains the active site. The flexible hinge of P-proteins orients the active site of RTA/Stx1A_1_ towards the SRL and in Step3 RTA/ Stx1A_1_ establish the specific contacts necessary to hydrolyze a single *N*-glycosidic bond in the SRL.

The arginine residues, which were critical for binding to the stalk proteins in Stx1A_1_ [73] and RTA [79] were on the opposite face of the active site, suggesting that both toxins interact with the ribosome in a similar manner. Analysis of the interaction of RTA with wild type and mutant yeast ribosomes deleted in stalk proteins by SPR showed that this interaction did not fit the 1:1 interaction model [81]. RTA interacted with wild type ribosomes by electrostatic interactions, which followed a two-step binding model. The two-step model is characterized by two different types of interactions with the ribosome, a saturable stalk dependent interaction with rapid association and dissociation rates and a much slower non-saturable stalk independent interaction with slower association and dissociation rates. The faster stalk dependent interaction was stronger than the slower stalk independent interaction. Further, the yeast mutant ribosomes lacking an intact stalk interacted with RTA by a 1:1 interaction model, which mirrored the slower interaction with wild type ribosomes [81]. According to the two-step interaction model shown schematically in Figure 2, in the first step RTA/Stx1A_1_ molecules are first concentrated on the surface of the ribosome via slow non-specific electrostatic interactions and are guided to the stalk. In the second step, rapid, more specific electrostatic interactions occur between the stalk binding surface of RTA/Stx1A_1_ and the CTD of the stalk proteins. In the third step, the P-protein stalk delivers RTA/Stx1A_1_ to the SRL by a conformational change of the flexible hinge region and allows RTA/Stx1A_1_ to depurinate the SRL at a very high rate. Consistent with this model, the interaction between RTA and the isolated native pentameric stalk complex from yeast fit well with a single step interaction model [89].

The enzyme coupled luminescence assay showed that the *K*_m_ values and catalytic rates (*k*_cat_) of the ribosome binding mutants of RTA for an SRL mimic RNA were similar to wild type RTA, indicating that their catalytic activity was not altered [79]. However, their *K_m_* was higher and their *k*_cat_ was lower towards ribosomes, indicating that the mutations affected ribosome binding and catalytic activity of RTA towards ribosomes without affecting RNA binding or catalytic activity of RTA towards naked RNA [79]. Based on this data, we proposed that arginines located on the opposite face of the active site of RTA bind to the flexible P-proteins of the ribosomal stalk. Stalk binding stimulates the catalysis of depurination by orienting the active site of RTA towards the SRL and thereby allows docking of the target adenine into the active site [79]. This model provided an explanation for why RTA depurinates intact ribosomes much better than free rRNA and how RTA hydrolyzes a single *N*-glycosidic bond on intact ribosomes from among the 4000 stem-loops in the large rRNA [79].

Subsequent studies showed that the ability to interact with the stalk was conserved in some RIPs, but not all RIPs [102]. PAP, which is a type-1 RIP active against ribosomes from all five kingdoms, interacts with ribosomal protein L3 to depurinate the SRL [103]. Since RTA, TCS and Stx1 were able to interact with the ribosomal stalk, was this ability to interact with the stalk a feature of an ancestral RIP, which has been conserved in some RIPs like ricin, Shiga toxins and TCS and lost in other RIPs like PAP? Phylogenetic analysis suggested that the ability to interact with the CTD of the ribosomal stalk arose independently in different RIPs by convergent evolution [104]. Further, the ability to interact with stalk was considered an adaptive advantage and did not have strong sequence constraints, which made it easy for different proteins to acquire this feature [104]. Based on the wide distribution of RIPs in plants and their presence in some bacteria, it has been postulated that an ancestral RIP domain present in plants was acquired by bacteria by horizontal gene transfer. However, a recent study presented evidence for the presence of RIP genes in Fungi and Metazoa and proposed that the differential loss of paralogous genes accounted for the complex pattern of RIP genes in extant species, rather than horizontal gene transfer [105].

Structural differences were shown between the structures of the CTD of bacterial and eukaryotic stalk proteins. The CTD of bacterial L12 is globular [106]. In contrast, NMR spectroscopy showed that while the *N*-terminal domain of eukaryotic P1/P2 dimer is structured, the CTD is flexible and can extend away from the dimerization region [107]. It has been suggested that these structural differences in the CTD may facilitate the domain specific recognition of elongation factors [20]. RTA is unable to depurinate intact *E. coli* ribosomes [82]. Similarly, TCS can only depurinate eukaryotic ribosomes, but not bacterial ribosomes. However, TCS was able to depurinate hybrid ribosomes when the bacterial stalk proteins were replaced with the eukaryotic stalk proteins [107]. These results suggested that the CTD and the flexible linker of stalk proteins are responsible for recruiting RIPs to the ribosome [107]. Therefore, RIPs like ricin and TCS that can only depurinate eukaryotic ribosomes may have evolved to bind to the CTD of eukaryotic stalk proteins, thereby hijacking the eukaryotic stalk proteins by binding to their *C*-terminal consensus sequences [20].

However, some critical questions remain. Stx1 can depurinate *E. coli* ribosomes, even though the stalk proteins differ in primary sequence and structure between the prokaryotes and the eukaryotes. Moreover, the conserved CTD of P proteins that can interact with Stx1 *in vitro*, is missing in the *E. coli* stalk proteins. Therefore, it is not clear how Stx1 accesses the SRL on *E. coli* ribosomes. Moreover, although the ribosome binding residues identified in Stx1A_1_ are conserved in Stx2A_1_, it is not known if they interact similarly with the ribosome. We have shown that there is a difference in the surface exposure of residues between Stx1A and Stx2A [70]. Arg176 is more exposed in Stx1A and is more critical for the depurination activity of Stx1A than Stx2A [70]. Arg176 has been shown to be important for binding of Stx1A_1_ to the ribosome [73]. It is not known if Arg176 has a similar role in binding of Stx2A_1_ to the ribosome. Although both Stx1A and Stx2A bind to the stalk, Stx2A is less dependent on the stalk proteins than Stx1A for its depurination activity [75]. These results indicate that there are differences in the ribosome interactions of Stx1 and Stx2, which may lead to differences in their depurination activity.

Evidence for structural differences between Stx1 and Stx2 and their importance in inactivation of the ribosome was obtained when Smith *et al.*, demonstrated that monoclonal antibody (MAb) 11E10, which neutralized both the cytotoxicity and lethality of Stx2, but not Stx1, bound to three specific regions around the active site of Stx2A, but failed to bind to Stx1A [108]. The sequence of the three regions was the most divergent between Stx2 and Stx1, which explained why the antibody specifically recognized Stx2 [108]. MAb 11E10 blocked the enzymatic activity of Stx2 *in vitro* and altered its intracellular trafficking pattern, providing evidence that structural differences lead to differential effects on the catalytic activity and trafficking of Stx1 and Stx2. Another MAb, S2C4, which was able to neutralize Stx2, but not Stx1 [109], was predicted to bind to another region that differed in sequence between Stx2 and Stx1 [82]. This region (residues 176–188) was shown to be important for binding of Stx1A_1_ to the ribosomal stalk [73], suggesting that structural differences between Stx1 and Stx2 may affect ribosome binding differentially. These results highlight the importance of identifying Stx2 residues, which are important in binding to the ribosome and the role of these residues on ribosome binding and depurination activity of each toxin.

Finally, in order for the toxin to depurinate the SRL specifically, it has to interact with the residues surrounding the SRL. Modeling analysis of the crystal structure of RTA and a 29-mer oligonucleotide hairpin containing the conserved GAGA loop of the SRL identified residues, which may be involved in binding to the 29-mer [110]. The amino acids at the active site are conserved between Stx1 and Stx2 [67,68,69,70,71,72]. However, there are conformational differences between the active sites of Stx1 and Stx2 [34,35] and the active site residues are more exposed in Stx2 than in Stx1 [70]. Currently it is not known if residues around the active site contribute to the catalytic activity of each toxin similarly. Analysis of depurination kinetics will shed more light on the relative role of these residues in binding to the SRL and in catalytic activity.

## 5. Conclusions

STEC are a serious cause of morbidity and mortality and a better understanding of their mechanism of virulence is of high significance. *In vivo* data indicate that the B subunits are involved in differential toxicity of Stx1 and Stx2, but do not rule out a potential role for the A subunits, suggesting that steps in addition to receptor binding and trafficking likely contribute to the differential toxicity of Stx1 and Stx2. The role of the A subunits in differential toxicity has not been fully examined. New discoveries indicate that the A subunits of Stx1 and Stx2 differ in their dependence on the ribosomal stalk proteins, suggesting that the role of the A subunits in ribosome binding, depurination activity and cytotoxicity may differ. Structural studies identified conformational differences in the active sites of the A subunits of Stx1 and Stx2. Monoclonal antibodies that selectively bind and neutralize Stx2 indicated important differences in enzymatic action and intracellular trafficking. A better understanding of the interaction of the A_1_ subunits of Stx1 and Stx2 with the ribosome and with the SRL, and comparative analyses of the catalysis of ribosome depurination are necessary to fully understand the factors that may contribute to the more pathogenic effects of Stx2 *in vivo*. These studies are relevant because they will have implications for the pathogenesis of HUS and may lead to the identification of novel therapeutic targets for Stx-associated HUS, for which there are no current therapies available.
